# Safety of esaxerenone (CS-3150) and its impacts on blood pressure and renal function: A systematic review and meta-analysis

**DOI:** 10.1097/MD.0000000000043615

**Published:** 2025-08-01

**Authors:** A.B.M. Kamrul-Hasan, Sunetra Mondal, Lakshmi Nagendra, Deep Dutta, Saptarshi Bhattacharya, Joseph M. Pappachan

**Affiliations:** a Department of Endocrinology, Mymensingh Medical College, Mymensingh, Bangladesh; b Department of Endocrinology, Nil Ratan Sircar (NRS) Medical College and Hospital, Kolkata, West Bengal, India; c Department of Endocrinology, JSS Medical College, JSS Academy of Higher Education and Research, Mysore, Karnataka, India; d Department of Endocrinology, CEDAR Superspeciality Healthcare, Dwarka, New Delhi, Delhi, India; e Department of Endocrinology, Indraprastha Apollo Hospitals, New Delhi, Delhi, India; f Faculty of Science, Manchester Metropolitan University, Manchester, United Kingdom; g Department of Endocrinology, Kasturba Medical College, Manipal Academy of Higher Education, Manipal, Karnataka, India.

**Keywords:** adverse events, albuminuria, esaxerenone, hyperkalemia, hypertension, mineralocorticoid receptor antagonists

## Abstract

**Background:**

The safety and efficacy of esaxerenone (ESAX), a novel nonsteroidal mineralocorticoid receptor antagonist, remain insufficiently explored in systematic reviews and meta-analyses (SR/MA). This SR/MA aimed to investigate the safety and effects of ESAX on blood pressure (BP) and renal function.

**Methods:**

Multiple databases and registers were systematically searched to identify randomized controlled trials and real-world studies evaluating the safety and efficacy of ESAX in various conditions. The primary outcome was the risk of adverse events (AEs); secondary outcomes included its effects on BP and renal parameters.

**Results:**

This SR/MA included 22 studies (N = 4699); 6 studies (5 randomized controlled trials and one retrospective study; n = 3211) with comparator groups were meta-analyzed. While more subjects on ESAX, especially at higher doses, experienced drug-related AEs (risk ratio [RR] 1.77) and discontinued due to these AEs (RR 6.75) compared to placebo, the number of subjects with any or serious AEs and drug-related serious AEs was similar between the 2 groups. Higher doses of ESAX were associated with increased risks of rising serum potassium levels (RR 3.30) and drug discontinuation related to these increases (RR 5.71) compared to the placebo. ESAX and active comparators exhibited comparable AEs except for a higher risk (RR 2.87) of increasing serum potassium levels with ESAX. ESAX led to larger decreases in estimated glomerular filtration rate and urine albumin-creatinine ratio than placebo. ESAX was more effective than placebo and active comparators in lowering office systolic and diastolic BP. ESAX 5 mg showed greater 24-hour average ambulatory BP reductions compared to the active comparators.

**Conclusion:**

ESAX appears reasonably safe, with a modest risk of hyperkalemia and worsening of renal function, and modest efficacy in the treatment of hypertension and albuminuria.

## 1. Introduction

Mineralocorticoid receptor (MR) antagonists (MRAs) are a well-established class of medications used to manage hypertension (HTN), particularly in hyperaldosteronism-related HTN.^[1]^ Some unique attributes of MRAs include their effectiveness in individuals with resistant HTN, prevention of cardiac remodeling,^[2]^ improvement of cardiovascular outcomes and reduction in cardiovascular mortality,^[3]^ decreased hospital admissions for heart failure (HF),^[4]^ reduction in renal proteinuria, slowing the progression of chronic kidney disease (CKD), particularly diabetic kidney disease,^[5,6]^ and lowering portal HTN in chronic liver disease.^[7]^ MRAs have been recognized to reduce cardiac fibrosis and decrease the recurrence of atrial fibrillation.^[8]^

Currently, spironolactone, eplerenone, and finerenone are the MRAs available for clinical use globally. Spironolactone is the oldest medication in the MRA class approved for clinical use. Aside from its relatively weak binding to the MR, its cross-reactivity with androgen receptors contributes to side effects such as menstrual irregularities in women and gynecomastia and impotence in men.^[9]^ Eplerenone and finerenone are highly selective MRAs. However, the issue of hyperkalemia has been observed with all 3 of these drugs, particularly in individuals with advanced CKD and when used together with angiotensin-converting enzyme inhibitors or angiotensin receptor blockers.^[9]^ Esaxerenone (ESAX) (CS-3150) has emerged as a novel and highly selective nonsteroidal MRA, demonstrating over 1000 times higher selectivity for the MR than other MR antagonists.^[10]^ Even at high doses, ESAX does not exhibit antagonism for androgen, progesterone, and glucocorticoid receptors.^[10,11]^ ESAX potently blocked the binding of [3H]-aldosterone to MR, with a median inhibitory concentration 50 of 9.4 nmol/L, better than eplerenone and spironolactone (inhibitory concentration 50 of 713 nmol/L and 36 nmol/L, respectively).^[11]^

Several randomized controlled trials (RCTs) and real-world studies have been published evaluating the use of ESAX in individuals with essential HTN, primary hyperaldosteronism (PA), diabetic and nondiabetic CKD, and in conjunction with other antihypertensive medications, and sodium-glucose cotransporter-2 inhibitors. In a meta-analysis of 3 RCTs involving individuals with essential HTN, ESAX 5 mg/day was found to be superior to ESAX 2.5 mg/day and eplerenone 50 mg/day in terms of reducing blood pressure (BP).^[12]^ However, the meta-analysis did not include all published RCTs of ESAX, particularly those conducted among subjects other than those with essential HTN. Furthermore, it failed to incorporate real-world studies with or without a non-ESAX control group. Thus, this systematic review and meta-analysis (SR/MA) of RCTs and real-world studies was conducted to establish a comprehensive understanding of the safety and optimal usage of this novel MRA in various clinical conditions.

## 2. Materials and methods

### 2.1. Ethical compliance

This SR/MA was conducted following the procedures described in the Cochrane Handbook for Systematic Reviews of Interventions and is reported according to the Preferred Reporting Items for Systematic Reviews and Meta-Analyses checklist.^[13,14]^ It has been registered with PROSPERO (CRD420251009636), and the protocol summary can be accessed online.

### 2.2. Search strategy

A systematic search was conducted across various databases and registries, including MEDLINE (via PubMed), Scopus, the Cochrane Library, ClinicalTrials.gov, and the Japan Registry of Clinical Trials. This search spanned from the inception of each database to February 5, 2025. Using the Boolean operators “AND” and “OR,” the following terms were searched: “esaxerenone,” “CS-3150,” “hypertension,” “essential hypertension,” “hyperaldosteronism,” “chronic kidney disease,” “albuminuria,” “diabetes mellitus,” “diabetic kidney disease.” The search terms were applied to titles and abstracts to identify both published and unpublished studies in the English language. Additionally, the search included reviewing references from the retrieved published articles and related journals.

### 2.3. Study selection

The Population, Intervention, Comparison, Outcomes, and Study design served as a framework to establish eligibility criteria for studies in this SR/MA. The patient population (P) consisted of adults of either sex with or without HTN or type 2 diabetes (T2D); the intervention (I) was ESAX either as monotherapy or combined with other antihypertensive medications; the comparison or control (C) group (optional) comprised individuals receiving a placebo or other active comparators either as monotherapy or in combination; and the outcomes (O) included safety and/or effectiveness of study drugs during the study period. RCTs or real-world studies (prospective or retrospective observational or intervention) were considered the study type (S) for inclusion. The studies with a comparator arm were included in the meta-analysis, whereas those without a non-ESAX control arm were used for the qualitative review. Exclusion criteria were case reports or case series, studies conducted in secondary HTN except for PA, studies undertaken in malignant HTN, studies conducted in type 1 or other types of diabetes, studies not reporting the outcomes of interest, and secondary or post hoc analysis of the included studies. Three review authors independently identified eligible articles according to the above criteria. Discrepancies in opinion on the inclusion of studies were resolved by consensus.

### 2.4. Data extraction and dealing with missing data

Three review authors independently extracted data using standardized forms; the details have been reported elsewhere.^[15]^ The following data were extracted for all the eligible studies and included in the review: first author, year of publication, trial registration number (if RCT), place of the study, study design, major inclusion and exclusion criteria of the study subjects, study arms, dose of ESAX and active comparators, sample size, the proportion of subjects with T2D and HTN, mean age, sitting systolic BP (SBP) and diastolic BP (DBP), 24-h average ambulatory SBP, 24-h average ambulatory DBP, proportions of subjects achieved target sitting BP of < 140/90 mm Hg, estimated glomerular filtration rate (eGFR), urine albumin-creatinine ratio (UACR), serum potassium (K^+^), brain natriuretic peptide (BNP) or N-terminal pro-brain natriuretic peptide (NTpBNP), and adverse events (AEs), including hyperkalemia. Any disagreements were resolved by consensus. The procedure for dealing with missing data has been reported elsewhere.^[15]^

### 2.5. Outcomes analyzed

The primary outcome of interest was the risk of AEs, including hyperkalemia, in the ESAX group compared to the control group. Additional outcomes included the changes from baseline in sitting SBP and DBP, 24-hour average ambulatory SBP and DBP, eGFR, percent UACR, serum K^+^, and proportions of subjects achieved target sitting BP of <140/90 mm Hg in the ESAX and control groups. The outcomes analysis was stratified based on the dose of ESAX and the type of control group, specifically a placebo, referred to as the placebo control group (PCG), or an active comparator, referred to as the active control group (ACG).

### 2.6. Statistical analysis

The results of the outcomes were reported using risk ratio (RR) for dichotomous variables and standardized mean difference (SMD) for continuous variables, together with 95% confidence intervals (CIs). The Review Manager computer program, version 7.2.0, was used to generate forest plots, which portrayed the odds ratio, RR, or SMD for the outcomes; the left side of the forest plot favored the ESAX group, and the right side favored the control group (PCG or ACG).^[16]^ Random-effects analysis models were chosen to address the anticipated heterogeneity resulting from variations in baseline population characteristics and study length. The inverse variance statistical method was applied for all instances. The SR/MA encompassed forest plots that integrated data from at least 2 trials. A significance level of *P* < .05 was used.

### 2.7. Assessment of the risk of bias in the included studies

Two authors independently assessed the risk of bias (RoB). The Cochrane risk-of-bias tool for randomized trials version 2 (RoB2) was used for assessing the RoB of the RCTs, whereas the Risk of Bias In Non-randomized Studies of Interventions version 2 (ROBINS-I V2) was used for non-randomized intervention trials and retrospective cohort studies.^[17,18]^ RoB2 and ROBINS-I V2 assessments have been described elsewhere.^[15]^ In discrepancies, the 6th and 7th authors acted as arbitrators to achieve consensus. The Risk-of-bias VISualization web app was used to generate RoB plots.^[19]^

### 2.8. Assessment of heterogeneity

The assessment of heterogeneity was initially conducted by studying forest plots. Subsequently, a Chi^2^ test was performed using N-1 degrees of freedom and a significance level of 0.05 to determine the statistical significance. The *I*^2^ test was also employed in the subsequent analysis.^[20]^ The specifics of understanding *I*^2^ values have been explained in depth elsewhere.^[15]^

## 3. Results

### 3.1. Search results

**Figure 1.**
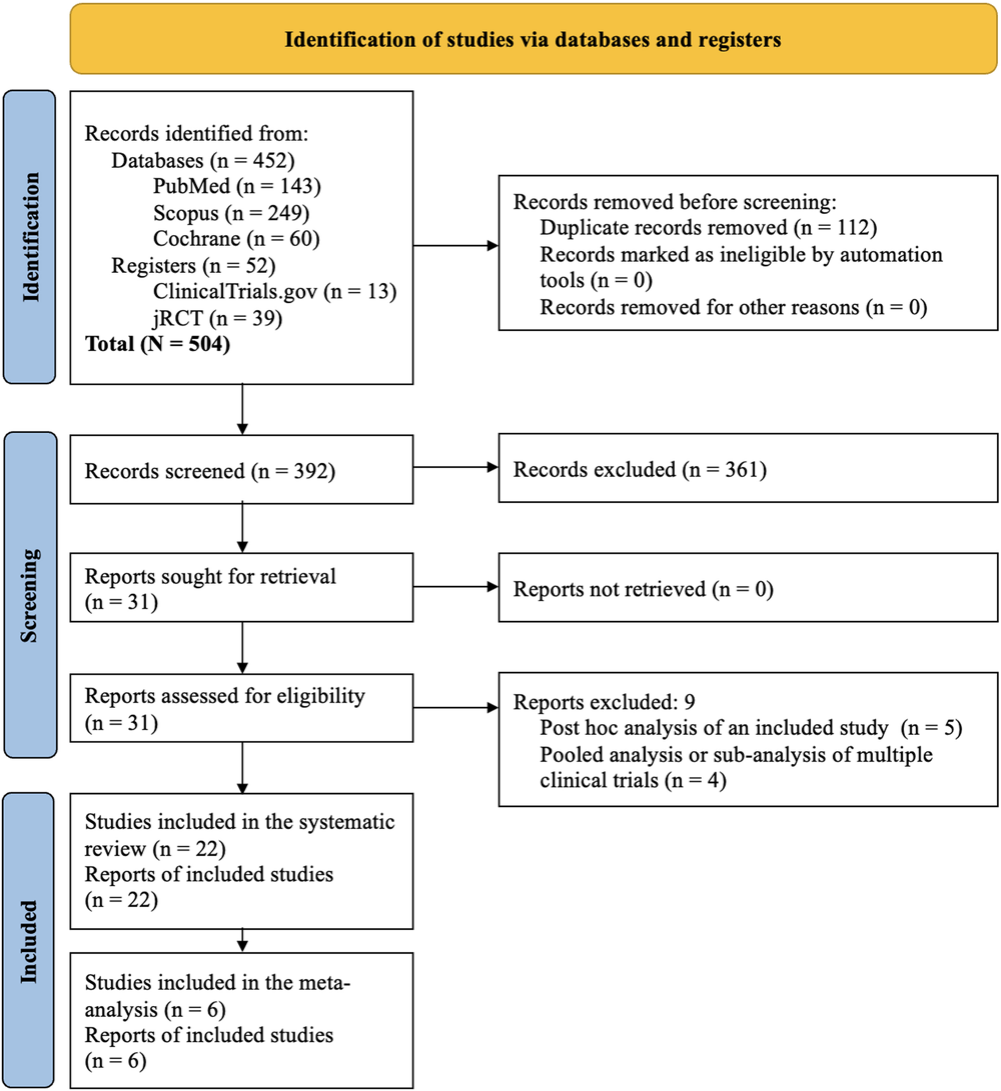
Flowchart on study retrieval and inclusion in the meta-analysis.

The Preferred Reporting Items for Systematic Reviews and Meta-Analyses flow diagram of steps in selecting the studies is depicted in Fig. 1. The initial search identified 504 articles, which were narrowed down to 31 after screening titles, abstracts, and subsequent full-text reviews. Finally, 22 studies (N = 4699) meeting all the prespecified criteria were included in the systematic review,^[21–42]^ of which 6 (n = 3211) were included in the meta-analysis.^[21–26]^ Nine studies were excluded,^[43–51]^ 5 were post hoc analyses of an included study,^[43–47]^ and 4 were pooled analyses or sub-analyses of >1 clinical trial.^[48–51]^

### 3.2. Characteristics of included and excluded studies

Table 1 summarizes the characteristics of the studies included in the meta-analysis. The specifics of studies included only in the systematic review and not in the meta-analysis are presented in Table 2. Five of the 6 studies were RCTs^[21–25]^; the other was a database-based retrospective cohort study.^[26]^ Two RCTs used a placebo in the control arm,^[22,23]^ 2 used an active comparator,^[24,25]^ and 1 RCT used both a placebo and an active comparator.^[21]^ Eplerenone was used in 2 RCTs,^[21,24]^ and trichlormethiazide was used in one as the active comparator.^[25]^ The included retrospective cohort study had 2 arms (one used ESAX in combination with other antihypertensive drugs, and the other arm used other antihypertensive medications without ESAX as mono- or combination therapy; this control arm was considered as the placebo-controlled arm in the meta-analysis).^[26]^ ESAX dose ranged from 0.625 to 5 mg/day in these studies. Four RCTs had durations of 12 weeks,^[21,22,24,25]^ while the other spanned 52 weeks.^[23]^ The retrospective cohort study lasted 36 months.^[26]^ The 16 studies included only in the systematic review (but not in the meta-analysis) lacked a non-ESAX control group.^[27–42]^ Twelve of these studies were prospective,^[27,29,30,32–36,38,39,41,42]^ while the other 4 were retrospective studies.^[28,31,37,40]^ Among these, 5 studies included study subjects with essential HTN,^[27–31]^ 4 included those with essential HTN and T2D,^[32–35]^ one included subjects with essential HTN and left ventricular hypertrophy,^[36]^ 2 included subjects with essential HTN and HF,^[37,38]^ one included those with nocturnal HTN,^[39]^ and the other 3 studies were conducted among individuals with primary hyperaldosteronism.^[40–42]^ Duration of these studies ranged from 12 to 52 weeks. The excluded studies have been summarized in Table S1, Supplemental Digital Content, https://links.lww.com/MD/P565.

**Table 1:** The characteristics of the studies included in the meta-analysis.

Trial ID, author name, registration no., type of study and phase, study place	Major characteristics of the study subjects	Study arms	N	T2D (%), HTN (%)	Age (y), mean (SD)	Sitting office SBP/DBP (mm Hg), mean (SD)	Serum K (mEq/L), mean (SD)	Duration
Ito 2019 (1), Ito et al, ^[21]^ jRCT2080222720, Phase 2 RCT, Multicenter in Japan	*Inclusion criteria:* Age ≥ 20 years, Sitting SBP ≥ 140 to <180 and DBP ≥ 90 to <110 mm Hg; and 24-h BP by ABPM ≥ 130/80 mm Hg. *Exclusion criteria:* Secondary or malignant HTN; diabetes with albuminuria; serum K^+^ <3.5 or ≥5.1 mEq/L; and eGFR <60 mL/min/1.73 m^2^.	Placebo	85	T2D 10.6, HTN 100	57.3 ± 9.1	157 ± 9/97 ± 5	4.14 ± 0.3	12 weeks
		Esaxerenone 1.25 mg/day	82	T2D 13.4, HTN 100	57.2 ± 9.3	156 ± 9/97 ± 5	4.07 ± 0.3	
		Esaxerenone 2.5 mg/day	84	T2D 9.5, HTN 100	56.8 ± 9.4	156 ± 8/99 ± 6	4.10 ± 0.3	
		Esaxerenone 5 mg/day	88	T2D 11.4, HTN 100	57.1 ± 8.8	157 ± 9/97 ± 5	4.14 ± 0.3	
		Eplerenone 50–100 mg/day	84	T2D 23.8, HTN 100	56.5 ± 10	158 ± 8/98 ± 5	4.09 ± 0.3	
Ito 2019 (2), Ito et al, ^[22]^ jRCT2080222722, JapicCTI-152774, NCT02345057, Phase 2 RCT, Multicenter in Japan	*Inclusion criteria:* Hypertensive or normotensive, T2D, age > 20 years, prior treatment with an RASi for at least 3 months, UACR 45 to <300 mg/g, and eGFR ≥ 30 mL/min/1.73 m^2^. *Exclusion criteria:* HbA1c ≥ 8.4%, non-DKD, secondary or malignant HTN, very high or low BP, hypokalemia or hyperkalemia.	Placebo	73	T2D 100, HTN 97	66 ± 10	138 ± 11/76 ± 9	4.3 ± 0.3	12 weeks
		Esaxerenone 0.625 mg/day	71	T2D 100, HTN 96	66 ± 9	138 ± 11/76 ± 9	4.2 ± 0.3	
		Esaxerenone 1.25 mg/day	72	T2D 100, HTN 97	66 ± 8	139 ± 10/76 ± 8	4.3 ± 0.3	
		Esaxerenone 2.5 mg/day	70	T2D 100, HTN 97	64 ± 11	137 ± 13/77 ± 9	4.3 ± 0.3	
		Esaxerenone 5 mg/day	72	T2D 100, HTN 94	65 ± 9	136 ± 12/76 ± 9	4.2 ± 0.3	
Ito 2020 (ESAX-DN), Ito et al, ^[23]^ jRCT2080223639, JapicCTI-173695, Phase 3 RCT, Multicenter in Japan	*Inclusion criteria:* Both HTN and T2D, age ≥ 20 years, prior treatment with an RASi for ≥12 weeks, UACR 45 to <300 mg/g, and eGFR ≥ 30 mL/min/1.73 m^2^. *Exclusion criteria:* HbA1c ≥ 8%, non-DKD, secondary or malignant HTN, very high or low BP, hypokalemia or hyperkalemia.	Placebo	227	T2D 100, HTN 100	66 ± 9	140 ± 10/84 ± 8	4.3 ± 0.3	52 weeks
		Esaxerenone 1.25–2.5 mg/day	222	T2D 100, HTN 100	66 ± 10	140 ± 10/83 ± 8	4.4 ± 0.3	
Ito 2020 (ESAX-HTN), Ito et al, ^[24]^ NCT02890173, jRCT2080223293, Phase 3 RCT, Multicenter in Japan	*Inclusion criteria:* Age ≥ 20 years, Sitting SBP 140–179 and DBP 90–109 mm Hg; and 24-h BP by ABPM ≥ 130/80 mm Hg. *Exclusion criteria:* Secondary or malignant HTN; diabetes with albuminuria; serum K^+^ <3.5 or ≥5.1 mEq/L; and eGFR <60 mL/min/1.73 m^2^.	Eplerenone 50 mg/day	331	T2D 18.4, HTN 100	55.8 ± 9.9	155.1 ± 9.6/98.3 ± 5.6	4.21 ± 0.3	12 weeks
		Esaxerenone 2.5 mg/day	330	T2D 14.8, HTN 100	55.9 ± 9.2	155.1 ± 9.6/98.1 ± 5.8	4.19 ± 0.3	
		Esaxerenone 5 mg/day	337	T2D 13.6, HTN 100	54.8 ± 9.7	155.6 ± 9.6/97.8 ± 5.4	4.22 ± 0.3	
Kario 2024 (EXCITE-HT), Kario et al, ^[25]^ jRCTs031220372, Phase 3 RCT, Multicenter in Japan	*Inclusion criteria:* Age ≥ 20 years, prior stable ARB or CCB for ≥4 weeks, mean morning home SBP ≥ 125 and/or DBP ≥ 75 mm Hg. *Exclusion criteria:* Secondary HTN; hyponatremia, hypokalemia or hyperkalemia; and eGFR <30 mL/min/1.73 m^2^, acute renal failure or anuria	Esaxerenone 2.5–5 mg/day	295	T2D 40.3, HTN 100	62.2 ± 11.5	144 ± 16/83 ± 12	4.21 ± 0.3	12 weeks
		Trichlormethiazide 0.25–3 mg/day	290	T2D 39.7, HTN 100	64.7 ± 12.1	143 ± 15/83 ± 12	4.21 ± 0.3	
Uchida 2025 (JDDM77), Uchida et al ^[26]^ Retrospective cohort study based on the Computerized Diabetic Care (CoDiC) database, Japan	*Inclusion criteria:* T2D with DKD and HTN	Esaxerenone 2.5–5 mg/day	199	T2D 100, HTN 100	67.24 ± 11.5	137 ± 16/75 ± 12	4.18 ± 0.52	36 months
		Non-esaxerenone	199	T2D 100, HTN 100	68.49 ± 11.34	135 ± 16/74 ± 11	4.29 ± 0.5	

ABPM = ambulatory blood pressure monitoring, ARB = angiotensin receptor blocker, BP = blood pressure, CCB = calcium channel blocker, DBP = diastolic blood pressure, DKD = diabetic kidney disease, eGFR = estimated glomerular filtration rate, HbA1c = glycated hemoglobin, HTN = hypertension, jRCT = Japan Registry of Clinical Trials, K^+^ = potassium, RASi = renin–angiotensin system inhibitor, SBP = systolic blood pressure, SD = standard deviation, T2D = type 2 diabetes, UACR = urine albumin-to-creatinine ratio.

**Table 2:** The characteristics of the studies included in the systematic review but not in the meta-analysis.

Authors, publication year	Major inclusion criteria	Study type (Reg. no.)	Duration	N	Age (years), mean ± SD or median [IQR]	∆Office SBP (mm Hg)	∆Office DBP (mm Hg)	∆UACR (mg/g or percent change)	∆eGFR (mL/min/1.73 m^2^)	eGFR decline > 20–30%) (%)	∆Serum K^+^ (mEq/L)	Serum K^+^ ≥ 5.5 mEq/L (%)	∆BNP or NTpBNP
Katsuya et al^[27]^ (ENaK Study)	Essential HTN on ARB or CCB	Exploratory, open-label, interventional (jRCTs031210273)	12 weeks	126	61.2 ± 11.6	‐12.3	‐7.9	‐37%	‐5.8	NR	0.1	7.1%	NTpBNP: −21.5%
Oshima et al^[28]^	Resistant essential HTN	Retrospective	24 weeks	26	70 [51–73]	‐12	‐3	−4 mg/g	2.3	NR	0.1	0	NR
Rakugi et al^[29]^	Essential HTN -untreated or on only one RASi or CCB	Open-label, optional dosage escalation, long-term phase 3 (NCT02722265	52 weeks	ESAX mono: 245	55.9 ± 9.4	−23.7	‐12.3	NR	‐6.3	NR	0.03	1.2	NTpBNP: −48%
				ESAX + CCB: 59	56.1 ± 8.9	‐20.5	‐13.1					0	NTpBNP: −45%
				ESAX + RASi: 64	57.2 ± 8.9	‐23.0	‐12.6					0	NTpBNP: −55%
Ito et al^[30]^	Essential HTN with eGFR 30–60 mL/min/1.73 m^2^	Single-arm, open-label, nonrandomized (NCT02448628)	12 weeks	ESAX mono: 33	63.9 ± 7.9	‐18.5	‐8.8	‐25.5%	−4.78	3 (↓ >30%)	0.3	0	NR
		Single-arm, open-label, nonrandomized (NCT02807987)	12 weeks	ESAX + RASi: 58	68 ± 7.7	‐17.8	‐8.1	‐28.5%	−4.45	5.2 (↓ >30%)	0.4	12.1	NR
Kitao et al^[31]^	Essential HTN with A1, A2 or A3 albuminuria	Retrospective	24 weeks	164 (A1 48, A2a 34, A2b 32, A3 50)	71 [60–78]	‐11*	2*	‐565 mg/g*	‐13*	NR	0.2	2.5	BNP: −72 pg/mL
Motoki et al^[32]^ (EAGLE-DH study)	Essential HTN, T2D on SGLT2i	Open-label, prospective, interventional	24 weeks	93	66.3 ± 9.9	‐11.8	‐7.1	‐49.1%	‐5.2	NR	0.07	1.1%	NR
Ito et al^[33]^	Essential HTN + T2D and UACR ≥ 300	Single-arm, open-label phase 3 (JapicCTI-173696)	28 weeks	56	65.7 ± 10.2	‐10.7	‐5.0	−54.6%	‐8.3	8.9 (↓ >30%)	NA	5.4	NR
Itoh et al^[34]^	Essential HTN + T2D and UACR 30-<1000	Single-arm, open-label	12 weeks	51	63 ± 9.8	−13.7	‐6.2	‐32.4%	‐4.5	NR	0.45	3.9	NR
Uchida et al^[35]^ (EX-DKD study)	Essential HTN with DKD	Open-label, Prospective (jRCTs061190027)	12 weeks	109	72.6 ± 7.0	‐11.5	‐5.2	‐50.9%	‐4.8	NR	0.29	2.7	NR
Yamamoto et al^[36]^ (ESES-LVH study)	Essential HTN with LVH, on RASi or CCB	Open-Label, prospective, Interventional (jRCTs071190043)	24 weeks	58	64.8 ± 12.7	‐15.1	‐6.5	NR	‐6.9	NR	0.23	5	NTpBNP: −13.9%
Iwahana et al^[37]^	Essential HTN with HFrEF (LVEF < 40%)	Retrospective, observational	Short-term: 35 ± 15 days, midterm: 167 ± 45 days	50	55.9 ± 15	Short-term: −7	Short-term: −3	NR	Short-term: ‐4.5, midterm: 3.78	Short-term: 14%, midterm: 16% (↓ >20%)	Short-term: 0.13, midterm: 0.21	Short-term: 11.8, midterm: 0	BNP: short-term: −112, midterm: −133 pg/mL
Naruke et al^[38]^	Resistant essential HTN with HF	Open-label, prospective, interventional	24 weeks	39	71 [65–79]	‐18†	‐7†	NR	NA	5.1 (↓ >30%)	NR	0	BNP: −73.9 pg/mL; no improvement in NYHA grade
Kario et al^[39]^ (EARLY-NH study)	Nocturnal HTN on ARB or CCB	Open-label, prospective interventional (jRCTs031200364)	12 weeks	101	67.6 ± 11.6	‐11.1	‐5.5	‐26.2%	‐5.5	NA	0.37	10.9%	NTpBNP: −18.5%
Fujimoto et al^[40]^	PA	Retrospective	9.1 weeks (median)	87	56 ± 11.9	‐5.6	‐2.8	‐51.5 mg/g	‐5.6	NR	0.11	2.3	BNP: −1 pg/mL
Satoh et al^[41]^	PA	Open-label, prospective, (NCT02885662; JapicCTI-163349)	12 weeks	44	49.6 ± 9.68	‐17.7	‐9.5	NR	‐8.1	4.5	0.33	4.5	NA
Yoshida et al^[42]^	PA	Prospective, Interventional	6 months	25	54 [49–66]	‐11	‐7	-3.6 mg/g	-10.2	NR	0.4	NR	NTpBNP: −6.4 pg/mL

ARB = angiotensin receptor blocker, BNP = brain natriuretic peptide, CCB = calcium channel blocker, DBP = diastolic blood pressure, DKD = diabetic kidney disease, eGFR = estimated glomerular filtration rate, ESAX = esaxerenone, HF = heart failure, HFrEF = heart failure with reduced ejection fraction, HTN = hypertension, jRCT = Japan Registry of Clinical Trials, K^+^ = potassium, LVEF = left ventricular ejection fraction, LVH = left ventricular hypertrophy, NR = data not reported, NTpBNP = N-terminal pro-brain natriuretic peptide, PA = primary hyperaldosteronism, RASi = renin angiotensin aldosterone system inhibitor, SBP = systolic blood pressure, SGLT2i = sodium-glucose cotransporter-2 inhibitors, T2D = Type 2 diabetes, UACR = urine albumin-to-creatinine ratio.

* In subgroup showing improvement in A3 to A2.

† Home BP.

### 3.3. Risk of bias of the included studies

Figure S1, Supplemental Digital Content, https://links.lww.com/MD/P566 depicts the RoB across the 22 studies included in the systematic review. The overall RoB, assessed by the RoB2 tool, was low in 4 of the 5 RCTs included in the meta-analysis; Kario 2024 (EXCITE-HT) had some bias concerns due to deviations from intended interventions (Fig. S1A, Supplemental Digital Content, https://links.lww.com/MD/P566). The non-randomized retrospective study, Uchida 2025 (JDDM77), included in the meta-analysis had a moderate overall RoB stemming from biases due to confounding, missing data, and in the selection of the reported result, as evaluated by the ROBINS-I assessment tool (Fig. S1B, Supplemental Digital Content, https://links.lww.com/MD/P566). Only one Kitao et al^[31]^ of the 16 non-randomized prospective and retrospective studies included in the systematic review (but not in the meta-analysis) had moderate overall RoB. The remaining 15 had serious RoB, primarily resulting from confounding bias, as evaluated by the ROBINS-I assessment tool (Fig. S1C, Supplemental Digital Content, https://links.lww.com/MD/P566). Evaluation of publication bias was not performed because there were insufficient RCTs (fewer than 10) in the forest plots.^[52]^

### 3.4. Adverse events

Identical proportions of study subjects in the ESAX and placebo groups had any AEs (RR 0.98, 95% CI [0.67, 1.42], *P* = .90) and serious AEs (RR 0.72, 95% CI [0.41, 1.26], *P* = .25). Although drug-related serious AEs were identical in the 2 groups (RR 0.76, 95% CI [0.03, 18.54], *P* = .87), higher proportions of subjects in the ESAX group had drug-related AEs (RR 1.77, 95% CI [1.02, 3.05], *P* = .04) and discontinuation of the study drug due to drug-related AEs (RR 6.75, 95% CI [1.83, 24.95], *P* = .004) than the placebo group. In subgroup analyses, risks of any AEs, serious AEs, and drug-related serious AEs were not increased with any of the ESAX doses. The increased risk of drug-related AEs occurred only with ESAX 5 mg, and discontinuation of the study drug because of drug-related AEs was noted with ESAX 2.5 mg and 5 mg (Table 3).

**Table 3:** Comparison of the safety outcomes in the esaxerenone versus placebo arms.

Outcome variables (categorical)	Esaxerenone dose	No. of participants with outcome/participants analyzed		Pooled effect size, RR (95% CI)	*I*^2^ (%)	*P*
		Esaxerenone arm	Placebo arm			
Any AE	All doses	450/767	257/388	0.99 [0.84, 1.17]	56	.90
	1.25 mg/day	75/155	80/159	0.92 [0.49, 1.73]	86	.80
	2.5 mg/day	258/380	257/388	1.03 [0.92, 1.15]	13	.62
	5 mg/day	79/161	80/159	0.98 [0.67, 1.42]	64	.90
Drug-related AE	All doses	118/767	32/388	1.77 [1.02, 3.05]	51	.04
	1.25 mg/day	19/155	16/159	1.22 [0.65, 2.28]	0	.54
	2.5 mg/day	60/380	32/388	1.56 [0.74, 3.28]	63	.24
	5 mg/day	33/161	16/159	2.03 [1.16, 3.55]	0	.01
Serious AE	All doses	22/767	26/388	0.72 [0.41, 1.26]	0	.25
	1.25 mg/day	2/155	2/159	0.98 [0.15, 6.60]	0	.98
	2.5 mg/day	19/380	26/388	0.76 [0.43, 1.33]	0	.34
	5 mg/day	1/161	2/159	0.73 [0.05, 10.31]	31	.82
Drug-related serious AE	All doses	1/767	0/388	0.76 [0.03, 18.54]	NA	.87
	1.25 mg/day	0/155	0/159	NE	NA	NA
	2.5 mg/day	1/380	0/388	3.08 [0.13, 74.46]	NA	.49
	5 mg/day	0/161	0/159	NE	NA	NA
Discontinuation of the study drug due to drug-related AE	All doses	27/767	2/388	6.75 [1.83, 24.95]	0	.004
	1.25 mg/day	1/155	0/159	3.00 [0.12, 72.44]	NA	.50
	2.5 mg/day	16/380	2/388	6.70 [1.79, 25.11]	0	.005
	5 mg/day	9/161	0/159	18.74 [1.11, 316.15]	NA	.04
Discontinuation of the study drug due to hyperkalemia	All doses	23/767	2/388	5.71 [1.36, 24.03]	0	.02
	1.25 mg/day	2/155	1/159	2.00 [0.19, 21.57]	NA	.57
	2.5 mg/day	12/380	2/388	5.14 [1.09, 24.25]	0	.04
	5 mg/day	7/161	1/159	6.90 [0.87, 54.71]	NA	.07
K^+^ increased	All doses	65/767	9/388	3.30 [1.30, 8.40]	38	.01
	1.25 mg/day	5/155	4/159	1.02 [0.10, 10.54]	50	.99
	2.5 mg/day	40/380	9/388	4.37 [2.12, 8.99]	0	<.0001
	5 mg/day	18/161	4/159	3.60 [0.75, 17.26]	48	.11
UA increased	All doses	12/541	2/159	1.76 [0.40, 7.77]	0	.46
	1.25 mg/day	5/155	2/159	2.54 [0.50, 12.96]	0	.26
	2.5 mg/day	1/154	2/159	0.64 [0.08, 5.18]	0	.68
	5 mg/day	5/161	2/159	2.45 [0.48, 12.52]	0	.28
eGFR decreased	All doses	13/481	4/316	2.27 [0.73, 7.02]	0	.16
	1.25 mg/day	3/83	1/87	3.14 [0.33, 29.63]	Na	.32
	2.5 mg/day	7/310	4/316	1.59 [0.35, 7.31]	16	.55
	5 mg/day	3/88	1/87	2.97 [0.31, 27.96]	NA	.34

AE = adverse events, CI = confidence interval, eGFR = estimated glomerular filtration rate, K^+^ = potassium, MD = mean difference, NA = not applicable, NE = not estimable, RR = risk ratio, UA = uric acid.

Identical proportions of study subjects in the ESAX group and the ACG experienced any AEs (RR 1.03, 95% CI [0.91, 1.26], *P* = .63), drug-related AEs (RR 1.11, 95% CI [0.58, 2.13], *P* = .75), serious AEs (RR 0.99, 95% CI [0.24, 4.11], *P* = .99), and discontinuation of the study drug due to drug-related AEs (RR 0.16, 95% CI [0.03, 0.98], *P* = .05). Subgroup analyses indicated comparable risks of these AEs with ESAX 2.5 mg and 5 mg compared to ACG. None of the study subjects in the ESAX group and ACG had drug-related serious AEs (Table 4).

**Table 4:** Comparison of the safety outcomes in the esaxerenone versus active comparator arms.

Outcome variables (categorical)	Esaxerenone dose	No. of participants with outcome/participants analyzed		Pooled effect size, RR (95% CI)	*I*^2^ (%)	*P*
		Esaxerenone arm	Active comparator arm			
Any AE	All doses	473/1226	266/714	1.03 [0.91, 1.16]	0	.63
	2.5 mg/day	161/415	154/416	1.05 [0.88, 1.25]	0	.60
	5 mg/day	287/728	266/714	1.05 [0.89, 1.25]	34	.56
Drug-related AE	All doses	101/1226	51/714	1.11 [0.58, 2.13]	69	.75
	2.5 mg/day	32/415	23/416	1.39 [0.83, 2.34]	0	.22
	5 mg/day	61/728	51/714	1.23 [0.58, 2.60]	74	.59
Serious AE	All doses	4/557	3/382	0.99 [0.24, 4.11]	0	.99
	2.5 mg/day	0/390	0/382	NE	NA	NA
	5 mg/day	3/390	3/382	0.99 [0.20, 4.85]	NA	.99
Drug-related serious AE	All doses	0/557	0/382	NE	NA	NA
	2.5 mg/day	0/84	0/84	NE	NA	NA
	5 mg/day	0/390	0/382	NE	NA	NA
Discontinuation of the study drug due to drug-related AE	All doses	1/557	6/382	0.16 [0.03, 0.98]	0	.05
	2.5 mg/day	0/84	1/84	0.33 [0.01, 8.07]	NA	.50
	5 mg/day	1/390	6/382	0.23 [0.04, 1.35]	0	.10
Discontinuation of the study drug due to hyperkalemia	All doses	0/255	0/84	NE	NA	NA
	2.5 mg/day	0/84	0/84	NE	NA	NA
	5 mg/day	0/88	0/84	NE	NA	NA
K^+^ increased	All doses	27/1226	4/714	2.87 [1.06, 7.79]	0	.04
	2.5 mg/day	11/415	4/416	2.75 [0.88, 8.58]	0	.08
	5 mg/day	16/728	4/714	3.06 [1.05, 8.93]	0	.04
UA increased	All doses	21/1226	7/714	1.38 [0.30, 6.39]	49	.68
	2.5 mg/day	8/415	3/416	2.59 [0.66, 10.07]	0	.17
	5 mg/day	11/728	7/714	1.48 [0.30, 7.41]	50	.63
eGFR decreased	All doses	7/557	3/382	1.02 [0.08, 12.45]	48	.99
	2.5 mg/day	0/84	0/84	NE	NA	NA
	5 mg/day	4/390	3/382	1.27 [0.07, 23.91]	60	.87

AE = adverse events, CI = confidence interval, eGFR = estimated glomerular filtration rate, K^+^ = potassium, MD = mean difference, NA = not applicable, NE = not estimable, RR = risk ratio, UA = uric acid.

### 3.5. Effect on serum K^+^

**Figure 2.**
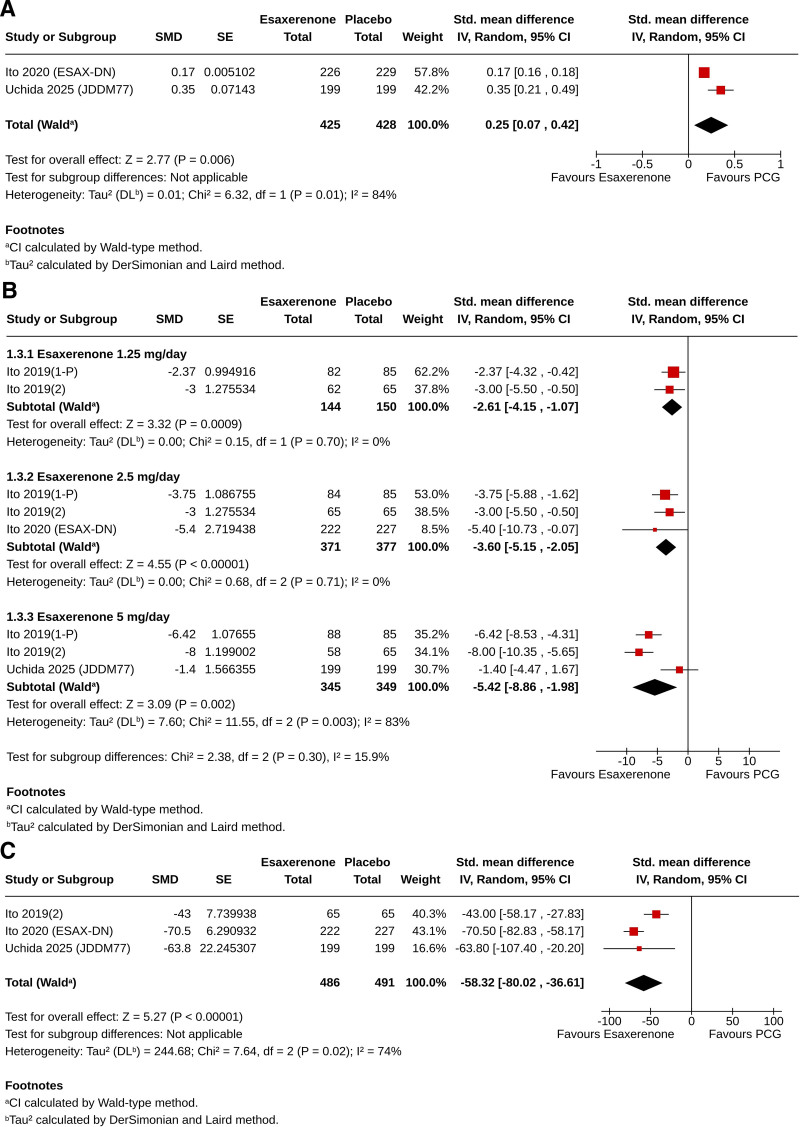
Forest plot showing the standardized mean differences of changes from baseline in the esaxerenone group compared to the placebo control group in – (A) serum potassium, (B) estimated glomerular filtration rate, and (C) urine albumin-to-creatinine ratio (percent).

In the meta-analysis, the increase in serum K^+^ from baseline was greater in the ESAX group than in the PCG (SMD 0.25, 95% CI [0.07, 0.42], *I*^2^ = 84%, *P* = .006) (Fig. 2A). A higher proportion of subjects in the ESAX group compared to PCG experienced an increase in serum K^+^ (RR 3.30, 95% CI [1.30, 8.40], *P* = .01) and discontinued the study drug due to hyperkalemia (RR 5.71, 95% CI [1.36, 24.03], *P* = .02). In both cases, higher risks were observed only for ESAX 2.5 mg compared to PCG (Table 3). In ACG-controlled trials, more subjects receiving ESAX experienced an increase in serum K^+^ levels compared to those on active comparators (RR 2.87, 95% CI [1.06, 7.79], *P* = .04). However, in the subgroup analysis, this risk was only observed with ESAX 5 mg compared to ACG. None of the participants in the ESAX group or the ACG discontinued the study drug due to hyperkalemia (Table 4).

The changes in serum K+ were highly variable in studies that lacked a non-ESAX control group in the systematic review. In patients with essential HTN, the increase in serum K^+^ from baseline ranged from 0.03 to 0.45 mEq/L; the rise was relatively greater (0.2–0.45 mEq/L) in studies involving patients with CKD. The increase in serum K^+^ in patients with PA ranged from 0.11 to 0.4 mEq/L. The rise in serum K^+^ levels of ≥5.5 mEq/L was reported in 0% to 11.8% of patients with essential HTN and 2.3% to 4.5% of patients with PA (Table 2).

### 3.6. Effect on eGFR

In the meta-analysis, greater reductions in eGFR were observed with all doses of ESAX compared to the placebo (for ESAX 1.25 mg: SMD −2.61, 95% CI [‐4.15, −1.07], *I*² = 0%, *P* = .0009; for ESAX 2.5 mg: SMD −3.6, 95% CI [‐5.15, −2.05], *I*² = 0%, *P* < .00001; and for ESAX 5 mg: SMD −5.42, 95% CI [‐8.86, −1.98], *I*² = 83%, *P* = .002) (Fig. 2B). eGFR decreased in comparable proportions of subjects in the ESAX versus PCG (RR 2.27, 95% CI [0.73, 7.02], *P* = .16) and ESAX versus ACG (RR 1.02, 95% CI [0.08, 12.45], *P* = .99). In subgroup analyses, similar risks of decreased eGFR were observed with ESAX 2.5 mg and 5 mg compared to PCG and ACG (Tables 3 and 4).

In noncontrolled studies, the change in eGFR from baseline ranged from 2.3 to −13 mL/min/1.73 m² in patients with essential HTN and from 5.6 to 10.2 mL/min/1.73 m² in those with PA. In these studies, 3% to 8.9% of participants experienced a decline in their baseline eGFR of more than 30% by the end. One study (Iwahana et al^[37]^) reported a 20% decline in eGFR among 14% of the subjects in the short term and 16% in the midterm (Table 2).

### 3.7. Effect on UACR

In the meta-analysis, a greater percentage reduction in UACR from baseline was observed with ESAX compared to the placebo (SMD −58.32, 95% CI [-80.02, −36.61], I² = 74%, *P* < .00001) (Fig. 2C). In noncontrolled studies, the absolute and percentage reductions in UACR ranged from 3.6 mg/g to 565 mg/g and 25.5% to 54.6%, respectively. The improvements in UACR were particularly notable in studies involving patients with T2D and albuminuria (Table 2).

### 3.8. Effect on serum uric acid

Serum uric acid (UA) increased in identical proportions of subjects in the ESAX versus PCG (RR 1.76, 95% CI [0.40, 7.77], *P* = .46) and ESAX versus ACG (RR 1.38, 95% CI [0.30, 6.69], *P* = .68). In subgroup analyses, similar risks of increased UA were observed with individual ESAX doses compared to PCG and ACG (Tables 3 and 4).

### 3.9. Effect on BP

**Figure 3.**
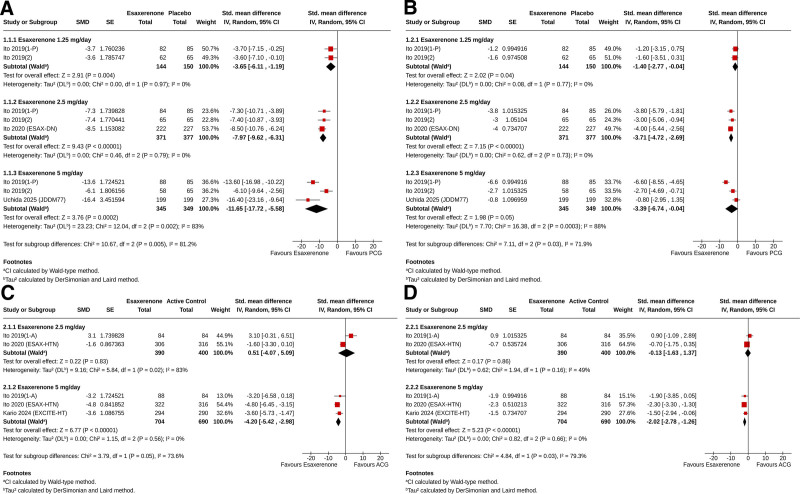
Forest plot showing the standardized mean differences of changes from baseline in – (A) systolic blood pressure and (B) diastolic blood pressure in the esaxerenone group compared to the placebo control group; and (C) systolic blood pressure and (D) diastolic blood pressure in the esaxerenone group compared to the active control group.

The meta-analysis showed that all doses of ESAX led to greater reductions in office SBP compared to placebo. Specifically, for ESAX 1.25 mg: SMD −3.65, 95% CI [‐6.11, −1.19], *I*² = 0%, *P* = .004; for 2.5 mg: SMD −7.97, 95% CI [‐9.62, −6.31], *I*² = 0%, *P* < .00001; and for 5 mg: SMD −11.65, 95% CI [‐17.72, −5.58], *I*² = 83%, *P* = .0002, as shown in Fig. 3A. Greater office DBP reduction was achieved with ESAX 1.25 mg (SMD −1.40, 95% CI [‐2.77, −0.04], *I*² = 0%, *P* = .04) and ESAX 2.5 mg (SMD −3.71, 95% CI [‐4.72, −2.69], *I*² = 0%, *P* < .00001), as well as with ESAX 5 mg (SMD −3.39, 95% CI [‐6.74, −0.04], *I*² = 88%, *P* = .05), compared to the placebo (Fig. 3B). Compared to ACG, the reduction in office SBP was similar with ESAX 2.5 mg (SMD 0.51, 95% CI [‐4.07, 5.09], *I*² = 83%, *P* = .83) and was greater with ESAX 5 mg (SMD −4.20, 95% CI [‐5.42, −2.98], *I*² = 0%, *P* < .00001) (Fig. 3C). Compared to ACG, the reduction in office DBP was identical with ESAX 2.5 mg (SMD −0.13, 95% CI [‐1.83, 1.37], *I*² = 49%, *P* = .86) and was greater with ESAX 5 mg (SMD −2.02, 95% CI [‐2.78, −1.26], *I*² = 0%, *P* < .00001) (Fig. 3D).

In noncontrolled studies, the changes in office SBP and DBP in patients with essential HTN ranged from −7 to −23.7 mm Hg and 2 to −12.3 mm Hg, respectively. In patients with PA, SBP and DBP decreased by 5.6 to 11.7 mm Hg and 2.8 to 9.5 mm Hg, respectively (Table 2).

### 3.10. Twenty-four hour average ambulatory systolic and diastolic BP

**Figure 4.**
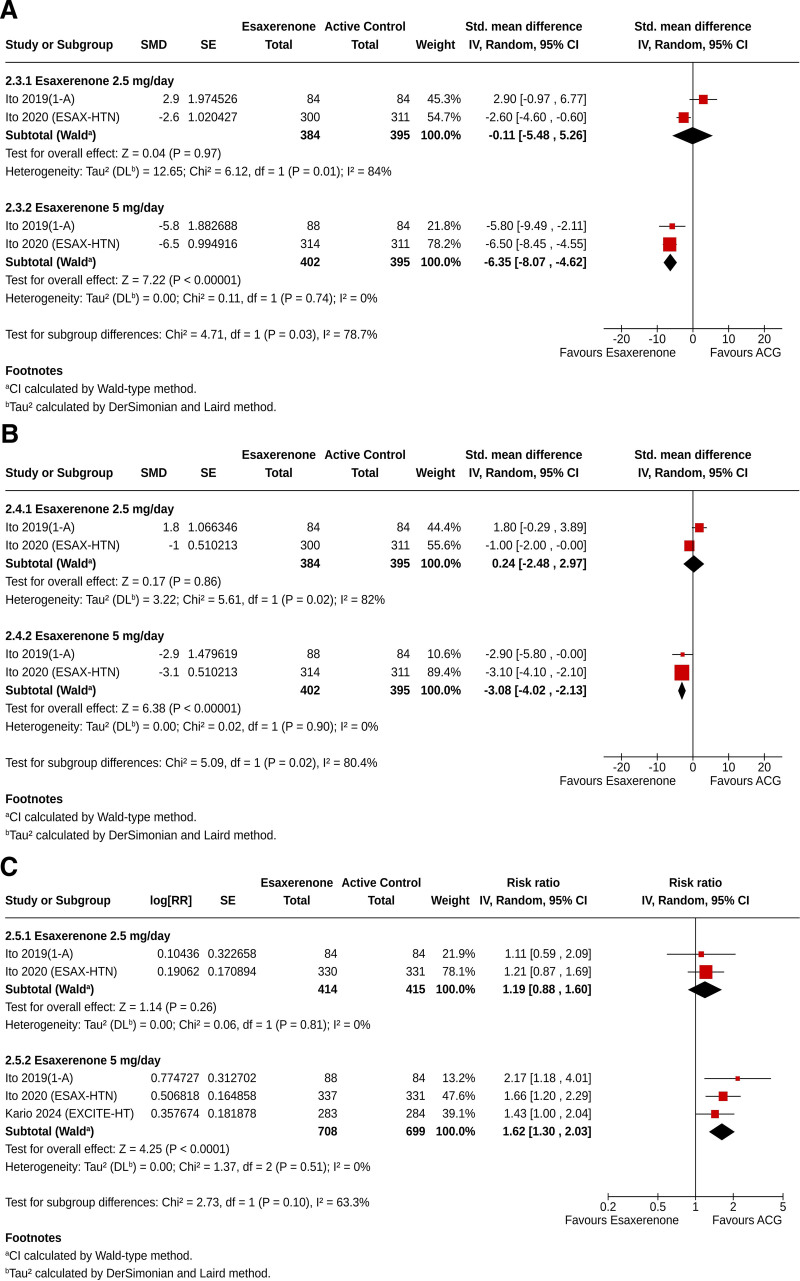
Forest plot showing the standardized mean differences of changes from baseline in – (A) 24-hour average ambulatory systolic blood pressure, (B) 24-hour average ambulatory diastolic blood pressure, and (C) proportions of subjects achieved target blood pressure of <140/90 mm Hg in the esaxerenone group compared to the active control group.

Compared to the ACG, ESAX 2.5 mg resulted in similar reductions in 24-hour average SBP (SMD −0.11, 95% CI [‐5.48, 5.26], *I*² = 84%, *P* = .97) and DBP (SMD −0.24, 95% CI [‐2.48, 2.97], *I*² = 82%, *P* = .86); however, greater reductions in 24-hour average SBP (SMD −6.35, 95% CI [‐8.07, −4.62], *I*² = 0%, *P* < .00001) and DBP (SMD −3.08, 95% CI [‐4.02, −2.13], *I*² = 0%, *P* < .00001) were observed with ESAX 5 mg (Fig. 4A and B).

### 3.11. Achievement of the target BP

Similar proportions of subjects receiving ESAX 2.5 mg achieved the target sitting BP of <140/90 mm Hg compared to ACG (RR 1.19, 95% CI [0.88, 1.60], *I*^2^ = 0%, *P* = .26); the proportion was higher with ESAX 5 mg than with ACG (RR 1.62, 95% CI [1.30, 2.03], *I*^2^ = 0%, *P* < .0001) (Fig. 4C).

### 3.12. Effect on BNP and NTpBNP

In the 2 studies involving patients with essential HTN and HF, the reduction in serum BNP was 133 pg/mL (Iwahana et al^[37]^) and 73.9 pg/mL (Naruke et al^[38]^); in patients without HF, reductions in NTproBNP ranged from 13.9% to 48%. The reductions in BNP and NTpBNP were minimal in PA (Table 2).

## 4. Discussion

### 4.1. Main findings of this review

This systematic review, involving 4699 participants from 22 studies, addressed the safety and efficacy of ESAX at different doses. Meta-analyses were conducted with only 6 studies, mostly with a low risk of bias, involving 3211 patients with comparator groups. Most of the 16 non-randomized studies assessed through qualitative synthesis only had serious risks of bias. Although a significantly higher proportion of participants experienced any drug-related AEs (RR 1.77) resulting in discontinuation (RR 6.75) of ESAX than with placebo (only at higher doses 2.5 and 5 mg), the rates of any AEs (RR 0.98), serious AEs (RR 0.72), and drug-related serious AEs (RR 0.76) were comparable between the drug and placebo, making ESAX a reasonably safe therapeutic agent. A significant increase in K^+^ levels from baseline compared to placebo was observed in a higher proportion of participants (RR 3.3), along with drug discontinuation due to hyperkalemia (RR 5.71) at doses of ≥2.5mg. Although the risk of hyperkalemia was higher (RR 2.87) only at a 5 mg dose of ESAX compared to active comparators, none of these cases required drug discontinuation. Although there was a modest yet significant dose-proportional reduction in eGFR ranging from −2.61 to 5.42 mL/min/1.73 m² with increasing doses of ESAX, no significant eGFR changes compared to placebo of ACG were observed. Similarly, no significant changes in serum UA were seen compared to PCG or ACG.

Regarding efficacy, a larger percentage reduction in UACR from baseline was observed with ESAX compared to placebo in the meta-analysis, especially in patients with T2D and albuminuria. While a greater and dose-proportional reduction of office SBP was observed with ESAX compared to placebo, the reduction was only seen with the 5 mg dose compared to ACG. Furthermore, greater reductions in office DBP were achieved with ESAX 1.25 mg, 2.5 mg, and 5 mg compared to placebo, while the largest reduction was observed with ESAX 5 mg compared to ACG. Greater reductions in 24-hour average SBP and DBP were observed with ESAX 5 mg compared to ACG. Additionally, more patients achieved target sitting BP < 140/90 mm Hg (RR 1.62) with ESAX 5 mg than with ACG. Reductions in BNP (73.9–133 pg/mL) were seen in 2 studies of patients with HTN and HF, and smaller reductions (13.9–48%) were observed in those without HF.

### 4.2. Implications for clinical practice

Although ESAX use was linked to a statistically significant increased risk of AE leading to drug discontinuation, only higher doses demonstrated this effect. Therefore, lower doses of the drug may still be used safely in clinical practice, considering the strong evidence supporting the use of MRA in patients with serious CVD such as HF.^[53–55]^ With its very high MR affinity and absence of androgenic side effects, ESAX is a highly effective alternative to spironolactone. As any AE, including hyperkalemia associated with the molecule, was comparable to ACG, including other nonsteroidal MRAs, except at a 5 mg dose, ESAX can be an effective alternative in patients with compelling indications. Although the drop in eGFR was similar to PCG and ACG in this review, special caution should be taken with patients who have advanced kidney disease, as the risk of acute kidney injury and hyperkalemia may be higher in these patients.^[5,56]^

A marked improvement in UACR associated with the drug has significant clinical implications, especially for high-risk patients like T2D cases, since albuminuria is recognized as the most serious risk factor for CVD and eGFR decline in these patients.^[57–59]^ Therefore, the timely institution of these molecules with disease-modifying properties should be considered in such patients. Significant improvements in HTN, including SBP, DBP, and 24-hour average SBP and DBP compared to placebo, make ESAX an appealing choice for resistant HTN and as an add-on therapy to other medications. This observation is especially important because every 5 mm Hg decrease in SBP is linked to a 10% reduction in major CV events.^[60]^ Furthermore, the ESAX 5 mg dose shows better efficacy compared to ACG, including a higher proportion of patients reaching target BP control. However, we should be cautious about potential excess AEs at higher doses. Significant reductions in SBP and DBP in patients with PA make this medication a viable alternative for those intolerant of spironolactone.

Significant reductions in BNP in patients with and without HF are also important. HF is often the end result of uncontrolled HTN, which is linked to a high risk of hospitalizations and increased mortality.^[60,61]^ Prompt reduction in BP and possibly other mechanisms, such as decreased cardiac fibrosis linked to MRA, might explain this observation, making ESAX an appealing new disease-modifying agent for patients with established HF and those at risk.

### 4.3. Strengths and limitations

Although the RoB was high among non-randomized studies in this review, the low RoB in most meta-analyzed studies strengthened our findings. Another important strength is the dose-specific comparisons we were able to perform regarding the efficacy and safety of ESAX in this study. However, we acknowledge the following limitations. For a very common disease like HTN, the total number of participants in the review (4699) and the meta-analysis (3211) is too small to draw firm conclusions about the efficacy and safety of the studied antihypertensive agent. Secondly, the study durations were relatively short (12 weeks to 36 months) for a lifelong condition like HTN, whose complications increase with the length of the disease. Therefore, the potential for cardiorenal protection associated with MRA use cannot be determined from our observations. Thirdly, the number of participants with HF and PA was relatively low, making it difficult to assess the efficacy and safety in these subgroups. Lastly, a cost-benefit analysis comparing this new molecule with other MRA agents was not feasible, and its high price might limit its use in resource-poor settings.

## 5. Conclusions

Based on small studies lasting from 12 weeks to 36 months, ESAX seems reasonably safe but shows a tendency for increased risk of drug-related AEs, including hyperkalemia and drug discontinuation at higher doses. ESAX also improves UACR and BP, with a higher proportion of participants reaching target BP control compared to placebo and ACG, especially at higher doses. Larger multinational, multiethnic RCTs with longer follow-up periods are necessary to assess the safety and effectiveness of ESAX and to establish appropriate clinical practice guidelines.

## Author contributions

**Conceptualization:** A.B.M. Kamrul-Hasan, Saptarshi Bhattacharya.

**Data curation:** A.B.M. Kamrul-Hasan, Sunetra Mondal.

**Formal analysis:** A.B.M. Kamrul-Hasan, Sunetra Mondal, Lakshmi Nagendra.

**Investigation:** A.B.M. Kamrul-Hasan, Deep Dutta, Saptarshi Bhattacharya, Joseph M. Pappachan.

**Methodology:** A.B.M. Kamrul-Hasan, Lakshmi Nagendra.

**Project administration:** A.B.M. Kamrul-Hasan, Deep Dutta, Joseph M. Pappachan.

**Resources:** A.B.M. Kamrul-Hasan, Sunetra Mondal, Lakshmi Nagendra, Deep Dutta, Saptarshi Bhattacharya.

**Software:** A.B.M. Kamrul-Hasan, Lakshmi Nagendra.

**Supervision:** Saptarshi Bhattacharya, Joseph M. Pappachan.

**Validation:** Deep Dutta, Saptarshi Bhattacharya, Joseph M. Pappachan.

**Visualization:** A.B.M. Kamrul-Hasan, Sunetra Mondal, Lakshmi Nagendra.

**Writing – original draft:** A.B.M. Kamrul-Hasan, Sunetra Mondal, Lakshmi Nagendra, Joseph M. Pappachan.

**Writing – review & editing:** A.B.M. Kamrul-Hasan, Deep Dutta, Saptarshi Bhattacharya, Joseph M. Pappachan.

## Supplementary Material


