# Supplementary material for: Safety of esaxerenone (CS-3150) and its impacts on blood pressure and renal function: A systematic review and meta-analysis
Source: Medicine (Baltimore). 2025 Aug 1;104(31):e43615. doi: 10.1097/MD.0000000000043615 (PMC12323939; doi:10.1097/MD.0000000000043615)
Supplement: Supplementary file 1 [file medi-104-e43615-s001.docx]

**Supplementary Table S1. Summary of the excluded studies.**

| **Authors, Publication year** | **Reason for exclusion** | **Study design** | **Summary of outcomes** |
| --- | --- | --- | --- |
| Ichikawa et al., 2022 [43] | Post hoc exploratory substudy of Rakugi et al., 2019 [29] | • Study subjects: essential HTN, an eGFR ≥60 mL/min/1.73 m^2^, and had not received any prior antihypertensive drug therapy or were receiving a CCB or RASi monotherapy  • Sample size: 25  • Intervention: esaxerenone monotherapy (2.5 or 5 mg/day) in treatment-naïve patients who continued the therapy during the 52-week study period | Esaxerenone monotherapy was associated with consistent reductions in SBP/DBP in the substudy population (- 23.5/- 13.1 mmHg) at week 52. |
| Kario et al., 2021 [44] | Post hoc analysis of ESAX-HTN trial [24] | • Study subjects: aged ≥20 years and uncontrolled essential HTN  • Sample size: 1001  • Intervention: Patients were randomized to esaxerenone 2.5 mg/day (n = 331) or 5 mg/day (n = 338), or eplerenone 50 mg/day (n = 332) for 12 weeks | • Greater reductions in nighttime SBP with 2.5 and 5 mg/day esaxerenone vs. eplerenone (−2.6 [−5.0, −0.2] and −6.4 mm Hg [−8.8, −4.0], respectively). .  • Such effects were greater in older patients and non-dippers. |
| Kario et al., 2022 [45] | Post hoc exploratory substudy of Rakugi et al., 2019 [29] | • Study subjects: essential HTN, an eGFR ≥60 mL/min/1.73 m^2^, and had not received any prior antihypertensive drug therapy or were receiving a CCB or RASi monotherapy  • Sample size: 368 (risers 53, non-dippers 162, dippers 123, and extreme dippers 29)  • Intervention: 59 received a concomitant CCB, 64 received a RAS inhibitor, and 245 received esaxerenone monotherapy (2.5 or 5 mg/day) for 28 weeks | • Nighttime systolic BP decreased in all dipping pattern groups at Week 28, with the riser group showing the greatest change (−25.5 mmHg).  • A significant shift in dipping pattern and riser/non-dipper pattern changes to dipper/extreme dipper pattern were found from baseline to week 28. |
| Kario et al., 2024(1) [46] | Post hoc analysis of  the EARLY-NH trial [39] | • Study subjects: aged ≥20 years and receiving a fixed dose of one ARB or one CCB  • Sample size: 82  • Intervention: Esaxerenone monotherapy (2.5 or 5 mg/day) for 12 weeks | • Among those who achieved target morning home SBP (<135 mmHg) and target bedtime home SBP (<135 mmHg), the brachial HBPM device showed achievement rates of 63.6% and 56.4%, respectively, for target nighttime home SBP (<120 mmHg).  • The wrist device showed achievement rates of 66.7% and 63.4%, respectively, for the same targets. Significant correlations were observed between both devices for nighttime home SBP measurements at baseline (r = 0.790), week 12 (r = 0.641), and change from baseline to week 12 (r = 0.533) (all, p < .001). |
| Okuda et al. 2023 [47] | Post hoc mediation analysis  of the ESAX-DN trial [23] | • Study subjects: Both HTN and T2D, age ≥20 years, prior treatment with an RASi for ≥12 weeks, UACR 45 to <300 mg/g, and eGFR ≥30 ml/min/1.73m^2^  • Sample size: 449  • Intervention: This post hoc analysis used a novel statistical method to quantitatively estimate the effect of esaxerenone on UACR reduction mediated, or not mediated, by changes in SBP and/or eGFR) | • The proportion of the mediated effect by SBP changes to the total effect on UACR reduction was 9.8–10.7%; the UACR was reduced to 0.903–0.911 times the baseline at the end of treatment through the SBP-related pathway and to 0.422–0.426 times the baseline through the non-SBP-related pathway.  • Even considering both SBP and eGFR simultaneously, the proportion of the mediated effect was 21.9–28.1%. |
| Ito et al., 2023 [48] | Pooled analysis of seven phase 3 trials | • Sample size: 1466 and 1472 in the full analysis and safety analysis sets, respectively  • Objective: Using pooled data from seven phase 3 studies, the study analysed factors associated with changes in office SBP and DBP from baseline to 12 weeks, and factors associated with incidence of serum K^+^ ≥5.5 mEq/L in esaxerenone-treated patients | • Male sex, weight ≥78.4 kg, HTN duration ≥10 years (2.66/1.71mmHg), prior antihypertensive treatment, plasma aldosterone concentration ≥120 pg/mL, UACR ≥300 or 30–299 mg/g, and smoking were associated with mean changes in SBP and DBP. • FPG ≥126 mg/dL was associated with the mean change in SBP only, and older age (65–74 years and ≥75 years, with mean change in DBP only.  • Factors significantly associated with incidence of serum K^+^ ≥5.5 mEq/L were higher baseline serum K^+^, higher UACR, higher DBP and grade I hypertension. |
| Kario et al., 2024(2) [49] | Pooled analysis of five clinical trials | • Sample size: 479 and 492 in the full analysis and safety analysis sets, respectively  • Objective: Aimed to identify factors associated with a strong home BP-lowering effect of esaxerenone and the incidence of elevated serum K^+^ in hypertensive patients | • Factors associated with a strong BP-lowering effect of esaxerenone were female sex and use of RASi as a basal antihypertensive drug.  • Patients with baseline serum K^+^ ≥4.5 mEq/L had an increased risk of developing elevated serum K^+^ ≥5.5 mEq/L after esaxerenone treatment. |
| Shikata et al., 2022 [50] | Sub-analysis of  two phase 3 trials | • Sample size: 505  • Objective: Changes in serum K^+^ levels during the studies and other measures were evaluated according to SGLT2 inhibitor use | • Concomitant use of SGLT2 inhibitor reduced the magnitude of serum K^+^ elevation without any change of its antihypertensive and albuminuria suppressing effects. |
| Yoshihara et al., 2024 [51] | Pooled analysis of five clinical trials | • Sample size: 1453  • Objective: The relationships between model-derived individual esaxerenone exposure and efficacy BP and safety (increased serum K+) were evaluated using multivariate linear regression and Cox regression analyses, respectively, using data from patients with or without diabetic kidney disease in five clinical studies. | Exposure-response analyses supported the esaxerenone recommended doses and the safety benefits of using the up-titration regimen. |

AGT, Angiotensinogen; ARB, Angiotensin II receptor blockers; CCB, Calcium-channel blocker; DSS, Dahl salt-sensitive; eGFR, Estimated glomerular filtration rate; FPG, Fasting plasma glucose; HBPM, Home blood pressure monitoring; HTN, Hypertension; K^+^, LVH, Left ventricular hypertrophy; Potassium; RASi, Renin-angiotensin system inhibitor; SBP, Systolic blood pressure; SGLT2, sodium-glucose co-transporter 2; UACR, Urinary albumin-to-creatinine ratio
