# Supplementary figures and images for: Safety of esaxerenone (CS-3150) and its impacts on blood pressure and renal function: A systematic review and meta-analysis
Source: Medicine (Baltimore). 2025 Aug 1;104(31):e43615. doi: 10.1097/MD.0000000000043615 (PMC12323939; doi:10.1097/MD.0000000000043615)

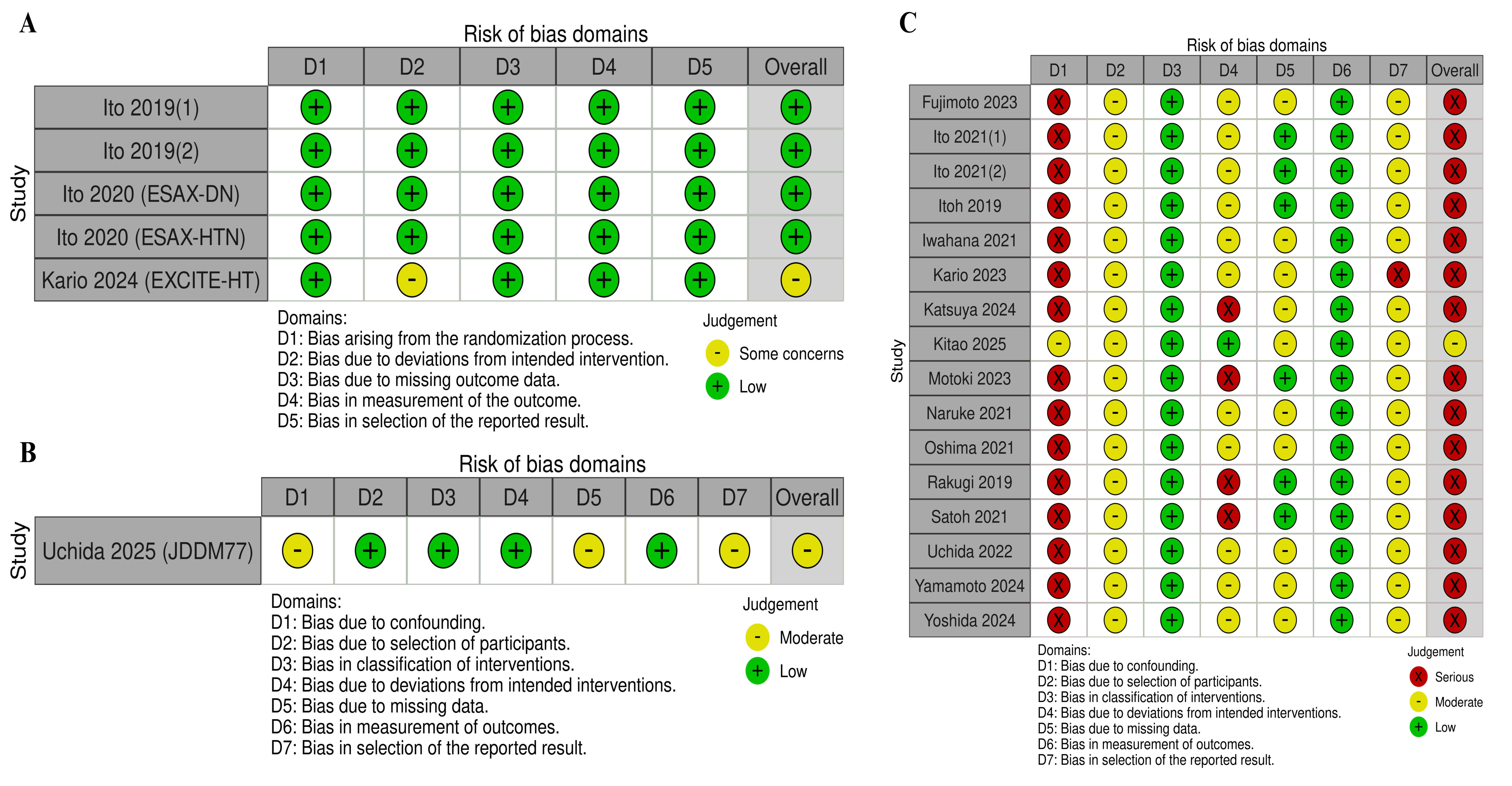

Supplement: Supplementary file 2 [file medi-104-e43615-s002.tiff]
